# An Uncommon Unilateral Grade V Thumb Hypoplasia: A Cadaveric Case Report

**DOI:** 10.7759/cureus.108262

**Published:** 2026-05-04

**Authors:** Jenna Farnum, Abby L Cummings, Tristan D Packard, Nathaniel G Blanchard, William McMillan, Libby J Bradley, Nicole L Geske

**Affiliations:** 1 Radiology, Division of Human Anatomy, College of Osteopathic Medicine, Michigan State University, East Lansing, USA

**Keywords:** abnormal anatomy, anatomy, cadaveric case report, prosection, thumb hypoplasia

## Abstract

Thumb hypoplasia is a rare congenital anomaly often associated with other syndromic manifestations. This case study explores a unilateral grade V thumb hypoplasia of a 97-year-old male anatomical donor with a medical history of cerebral atherosclerosis, dementia, hypertension, benign prostatic hyperplasia, and a transient ischemic attack. A thorough dissection of the forearm and hand confirmed a suspected grade V thumb hypoplasia and atypical surrounding anatomy. This study contributes to the literature regarding thumb hypoplasia and discusses potential complications and areas of concern for related surgical procedures, congenital syndromes, or additional body-wide manifestations.

## Introduction

Thumb hypoplasia is an uncommon congenital anomaly that can present in varying degrees of anatomical underdevelopment and functional impairment. The reported incidence is about one in every 100,000 live births, with an equal distribution between men and women [1]. Although classification systems have been published to guide clinical management, there is limited published literature detailing the spectrum of rare or atypical presentations. As a result, unusual structural findings may be underrecognized despite their relevance to understanding functional impairment, diagnosis, and management of thumb hypoplasia.

The clinical and practical importance of the thumb cannot be overstated, particularly given its central role in fine motor coordination and overall grip strength. As a defining anatomical feature of the human hand, the thumb is essential for object manipulation and precision tasks. Its unique ability to oppose the fingers enables precision grip, which is required for many daily activities such as writing, buttoning clothing, and using tools. Congenital absence (aplasia) or hypoplasia of the thumb results in the loss of opposition, leading to significant impairment in grasp, pinch strength, and manual dexterity. Individuals affected by this condition often struggle with basic tasks that require fine motor control, including tying shoelaces, opening containers, and handling small objects.

Where thumb aplasia refers to the complete absence of the thumb, hypoplasia refers to the underdevelopment of the thumb, and its severity is most commonly described using the Blauth classification [2]. In Grade I hypoplasia, the thumb is present but smaller than normal, with only mild functional impairment [3-5]. Grade II is characterized by narrowing or instability of the first metacarpal and metacarpophalangeal joint, along with weakness of the thenar muscles [3-5]. Grade III involves partial absence of skeletal elements, such as a markedly deficient first metacarpal, resulting in limited or absent opposition and significant functional compromise [3-5]. In Grade IV, often referred to as a “floating thumb,” only a rudimentary soft tissue remnant remains with little to no function. Grade V represents a complete absence of the thumb at the opponens position, including musculature and skeletal elements [3-5]. Grade V is used to reference aplasia, in which the thumb is absent. The Blauth classification not only provides a framework for describing the degree of hypoplasia but also guides management, with milder forms (Grades I-II) often treated with reconstructive procedures such as opponensplasty, while more severe forms (Grades III-V) typically require pollicization or other surgical reconstruction to restore hand function [3-5].

Abnormal neurovascular anatomy has been consistently reported in cases of thumb hypoplasia and radial-longitudinal deficiency. Early angiographic studies demonstrated that the radial artery is absent or severely hypoplastic in the majority of cases of radial artery deficiency, with the median artery, a rare remnant of the fetal circulation, frequently persisting to supply the hand [6]. In some patients, the thumb is perfused entirely by branches of the superficial palmar arch, with the princeps pollicis and radialis indicis arteries arising from the ulnar artery rather than the radial artery [7]. In addition, surgical and anatomical case reports describe a single midline neurovascular bundle in hypoplastic thumbs, replacing the normal paired digital neurovascular structures and often associated with absent thenar musculature [8]. Other reports highlight variant arterial pedicles around the index finger and first web space in the setting of congenital thumb hypoplasia, underscoring the variability of inflow patterns [9]. Finally, literature on congenital thenar hypoplasia (Cavanagh syndrome) notes accompanying anomalies of the median nerve and regional vasculature, further supporting the close relationship between neurovascular and musculoskeletal deficiencies in these patients [10].

Grade V thumb hypoplasia or aplasia may occur as an isolated anomaly or as part of a broader syndromic condition. Several congenital syndromes are frequently associated with thumb anomalies. Holt-Oram syndrome is characterized by malformations of the upper limbs, including thumb hypoplasia or aplasia, in conjunction with congenital cardiac defects [11]. Fanconi anemia presents with radial ray anomalies alongside hematologic abnormalities such as pancytopenia and bone marrow failure [11]. Additionally, VACTERL association (Vertebral defects, Anal atresia, Cardiac defects, Tracheo-esophageal fistula, Esophageal atresia, Renal (kidney) anomalies, and Limb abnormalities) includes a constellation of anomalies-vertebral, anal, cardiac, tracheoesophageal, renal, and limb abnormalities, with radial and thumb deficiencies being common findings [11]. Radial dysplasia, or radial longitudinal deficiency, is a term used to describe the spectrum of congenital anomalies primarily affecting the thumb, carpus, and radius, ranging from hypoplasia to aplasia [12]. The spectrum of these disorders can reflect many additional systemic findings; therefore, upon identification of a patient with suspected congenital thumb hypoplasia, a thorough workup should be considered. Overall, embryologic and genetic causes of radial dysplasia are largely unknown but are thought to be a result of sonic hedgehog gene mutations. Other hypotheses include teratogenic exposures, vascular insufficiency, and intrauterine or environmental factors [12].

Despite the functional challenges associated with thumb hypoplasia, several therapeutic and surgical interventions can enhance one’s quality of life. Management options include occupational therapy, thumb reconstruction procedures, and index finger pollicization - a surgical technique in which the index finger is repositioned to function as a thumb [13]. In this case study, the donor presented with Type V thumb aplasia, an index pollicization would likely have been the recommended approach, as it typically offers the most significant functional improvement in severe cases [13]. This study will contribute to the existing understanding of thumb hypoplasia observed in cadaveric sources and provide a thorough description of the observed anatomy that deviates from the expected.

## Case presentation

During routine dissection of an anatomical donor, conducted in accordance with the Michigan State University Division of Human Anatomy’s standard prosection protocol for the upper extremity, it was observed that the left thumb was absent. Typical anatomy was noted on the right upper limb of the donor; however, the donor was no longer available for further research at the time of this investigation. Further investigative dissection was performed to determine if the anatomy supported a congenital or acquired etiology.

On the posterior forearm, extensor pollicis longus and extensor indicis and their corresponding tendons traveled adjacently through a tendon sheath. Tendons were traced from their insertions to their corresponding muscle bellies for identification, with reference to the surrounding normal anatomy of the forearm.

**Figure 1:**
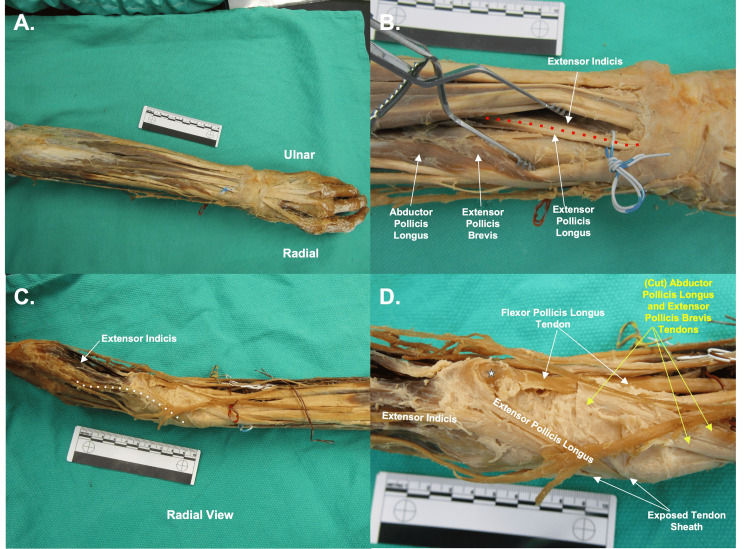
Posterior Forearm Panel A: Overview of posterior forearm anatomy. Blue and white dashed wire represents the tendons of extensor indicis and extensor pollicis longus. Panel B: Close-up view of the extensor indicis and extensor pollicis longus muscles. Red dotted line indicates the delineation of the extensor indicis and pollicis longus tendons. Panel C: Radial side view of forearm. Demonstrates extensor indicis tendon traveling toward index after emerging from the shared tendon sheath. Panel D: Resected shared tendon sheath of extensor indicis and extensor pollicis longus. Extensor pollicis longus inserts onto the base of the second metacarpal (indicated by white star).

As observed in Figure 1, once these tendons exited this sheath, the extensor indicis resumed its normal route towards the extensor hood of the index, whereas the extensor pollicis longus inserted onto the base of the second metacarpal. The tendons of extensor pollicis brevis and abductor pollicis brevis wrapped around the extensor carpi radialis longus and brevis tendons as expected, but their insertions appeared to adhere to the flexor tendon sheath.

**Figure 2:**
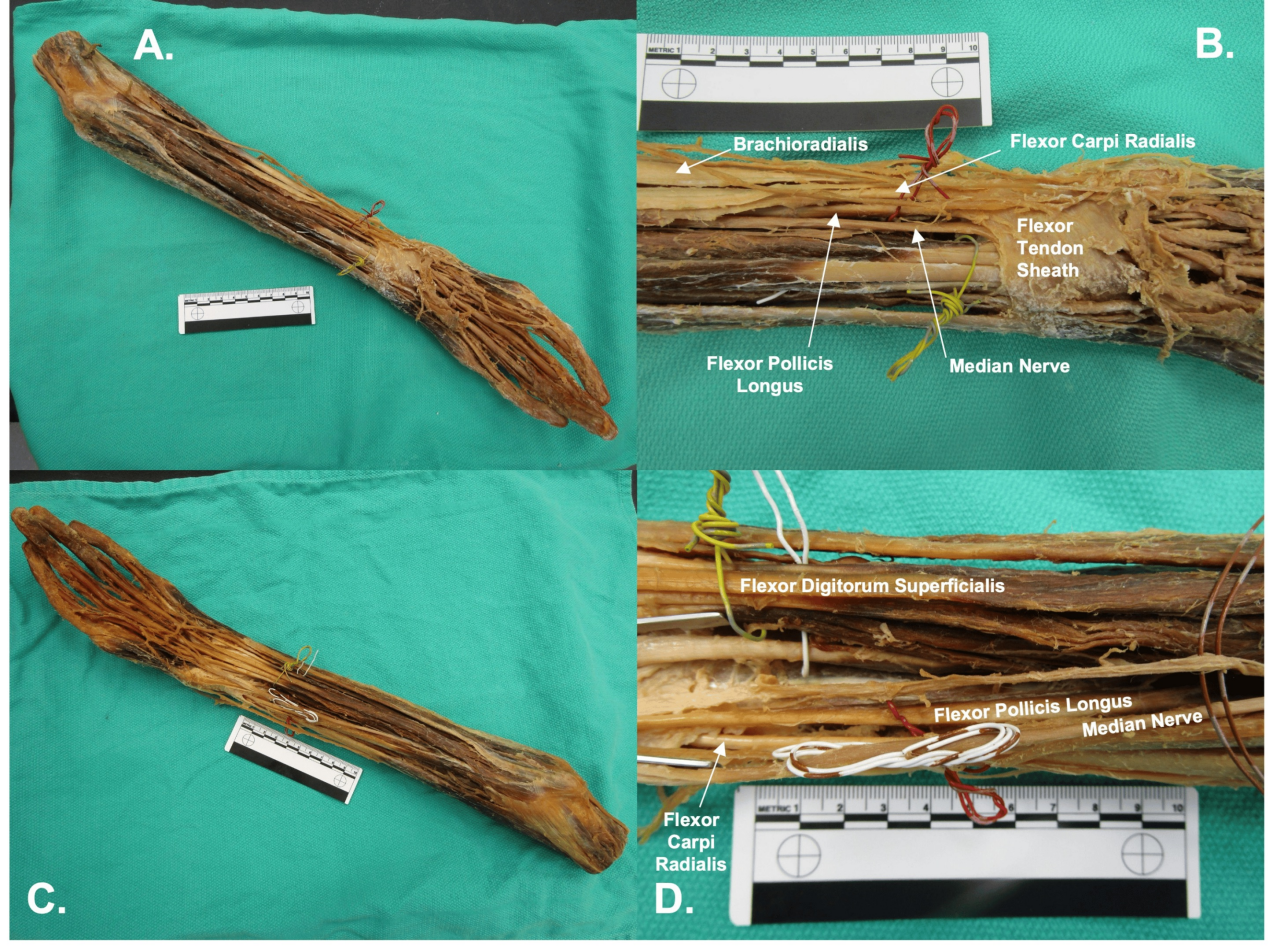
Anterior Forearm Panel A: Forearm overview with flexor tendon sheath intact. Panel B: Close-up of the distal forearm, with proposed flexor pollicis longus tendon, flexor carpi radialis tendon, and median nerve, with flexor tendon sheath intact. Panel C: Forearm overview with flexor tendon sheath removed. Panel D:  Close-up of the distal forearm with flexor digitorum superficialis tendons, flexor pollicis longus tendon, flexor carpi radialis tendon, and the median nerve.

As observed in Figure 2, the flexor pollicis longus tendon merged with the flexor retinaculum. Passing through the carpal tunnel were the four tendons of flexor digitorum superficialis, the four tendons of flexor digitorum profundus, the flexor pollicis longus tendon, and the median nerve. However, a small branch of the radial artery adhered to the median nerve. This branch of the radial artery variably measured 0.2-0.4mm in diameter. Another small branch of the radial artery was also present in its typical location along the radial aspect of the anterior forearm. It is likely that the branch of the radial artery found with the median nerve was at one time a branch of the radial artery, but that relationship was lost during dissection. The radial artery found along the radial aspect of the anterior forearm variably measured 0.2-0.3 mm in diameter. This artery was traced along the radial aspect of the hand. It was not present in the anatomical snuffbox and instead narrowed to 0.1 mm in diameter, contributing to the deep palmar arch.

The branch of the radial artery that traveled through the carpal tunnel with the median nerve contributed to the superficial palmar arch, along with the ulnar artery. The ulnar artery was identified in its typical location, passing through the ulnar canal and measuring 0.4 mm in diameter.

**Figure 3:**
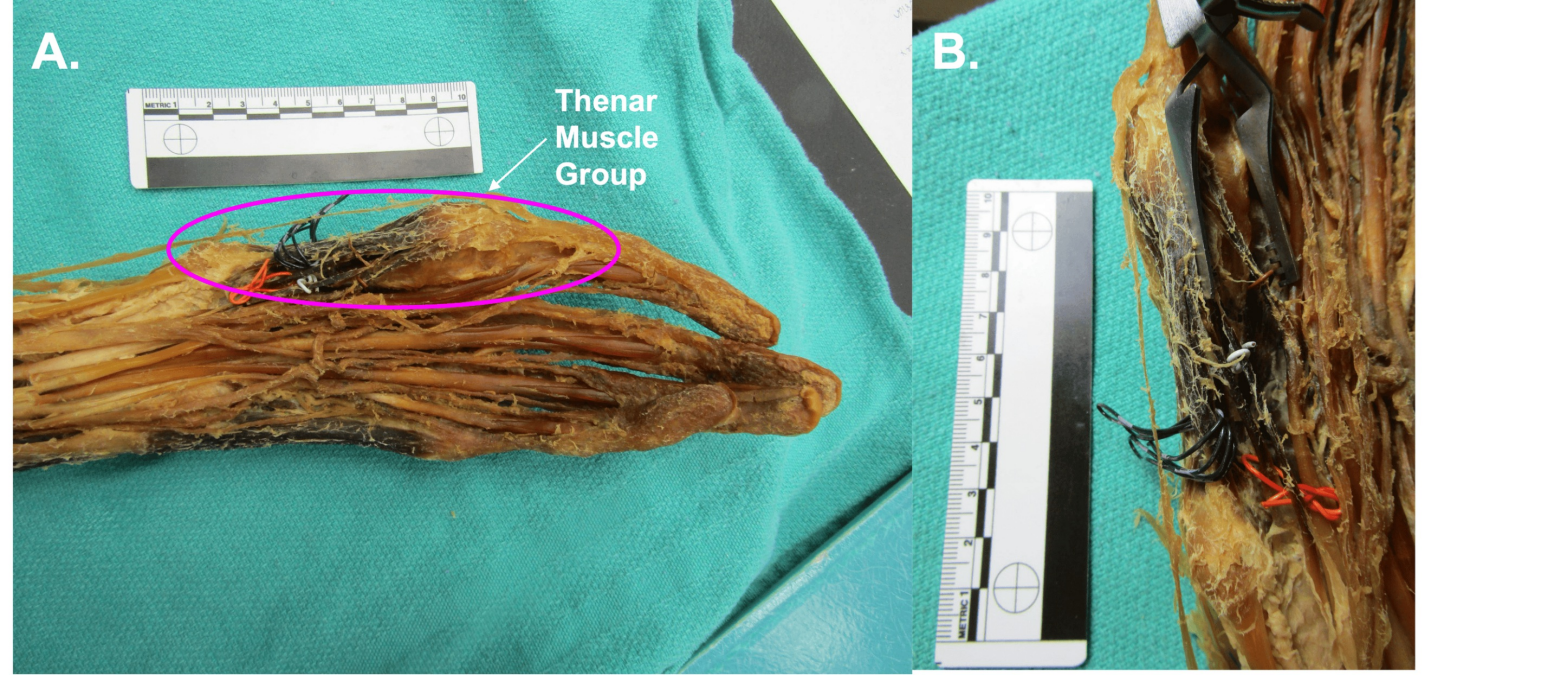
Palmar Anatomy Panel A: Overview of hypothesized thenar muscle group. Panel B: Black wire, red wire, and white wire distinguish separate muscle bellies of the proposed thenar muscle groups.

As observed in Figure 3, within the hand, a hypothesized remnant of the thenar muscles is attached to the carpal bones and the distal portion of the second metacarpal. Due to the significantly altered anatomy, the authors refrained from identifying these muscle bellies individually, but hypothesize that they do correspond to remnant thenar muscles (abductor pollicis brevis, opponens pollicis, and flexor pollicis brevis).

## Discussion

The donor's thumb aplasia was accompanied by muscular and vascular abnormalities in the forearm, carpal region, and hand. Typically, the radial artery is a prominent, palpable structure that passes through the anatomical snuff box; however, in this case study, a small branch of the radial artery was adhered to the median nerve and passed through the carpal tunnel. This branch of the radial artery contributed to the superficial palmar arch. An additional branch of the radial artery coursed along the radial aspect of the anterior forearm, significantly narrowed, and contributed to the deep palmar arch. Both radial arteries were small in diameter, which is consistent with the literature, in which radial artery hypoplasia is commonly reported with cases of thumb hypoplasia [11]. Radial artery hypoplasia or absence is well documented in cases of radial longitudinal deficiency and thumb hypoplasia, and in compensation, there is enlargement of the ulnar artery and reliance on the superficial palmar arch [14,15].

The extensor pollicis longus typically inserts on the base of the first distal phalanx. However, in this donor, the extensor pollicis longus muscle is inserted on the base of the second metacarpal. Additionally, the extensor pollicis brevis and abductor pollicis longus are typically small muscles that curve around the posterior distal portion of the radius and insert on the first proximal phalanx and first metacarpal, respectively. The attachment sites are crucial to extension and abduction of the thumb [1]. Anomalous connections between thumb and index extensor tendons have previously been described, suggesting that in the absence of normal thumb anatomy, tendon structures may be redirected toward adjacent digits [16-18]. The tendons of extensor pollicis brevis and abductor pollicis longus in this donor wrapped around the extensor carpi radialis longus and brevis tendons as expected, but their insertions instead appeared to adhere to the flexor tendon sheath. Variations in abductor pollicis longus and extensor pollicis brevis insertions are well documented, including multiple tendon slips and variable insertion sites onto the metacarpal, trapezium, or surrounding soft tissues [19,20]. While convergence with the flexor tendon sheath has not been previously described, the degree of variability reported in these tendons suggests that altered insertion patterns may occur in severe thumb hypoplasia. It is unlikely that extensor pollicis longus, extensor pollicis brevis, and abductor pollicis longus had any significant contributions to the second digit in this individual, although it is possible that there may have been some additional abduction and extension of this digit beyond the typical.

The thumb is usually supported by three intrinsic hand muscles, known collectively as the thenar muscles: the abductor pollicis brevis, opponens pollicis, and flexor pollicis brevis. The opponens pollicis originates on the trapezium and flexor retinaculum and inserts on the first metacarpal. The abductor pollicis brevis originates from the scaphoid, trapezium, and flexor retinaculum and inserts on the base of the first proximal phalanx. The flexor pollicis brevis originates on the trapezium and the flexor retinaculum and also inserts on the first proximal phalanx [21]. As observed in this donor, the thenar muscles are significantly reduced in breadth and appear to insert on the distal portion of the second metacarpal. It is unlikely that the thenar musculature contributed significantly to abduction or flexion of the second digit based on its size.

As there was no evidence of surgical scarring, it was determined that the thumb aplasia is congenital. Congenital thumb hypoplasia may be associated with more serious implications than altered thumb mechanics alone, as it can signal underlying developmental abnormalities involving the cardiac, renal, or hematologic systems. These conditions may include syndromes such as VACTERL, Holt-Oram syndrome, thrombocytopenia absent radius (TAR), and Fanconi anemia. Therefore, clinicians should remain aware of the potential association between thumb hypoplasia/aplasia and broader multisystem disorders, and exercise appropriate clinical judgment to investigate these possibilities when warranted.

In procedures such as pollicization, understanding the course and insertion of anomalous tendons is important for planning reconstruction in order to optimize functional outcomes. In pollicization procedures, there is a redirection of thumb-associated tendons toward the remaining index finger to permit an increase in range of motion and thumb-like movements [1]. An additional implication that is potentially relevant to other in vivo examples of grade V thumb hypoplasia (aplasia) is the hypoplasia of the radial artery and its compensatory proximity to the median nerve, raising concerns about vascular preservation and the potential risk of nerve injury during surgical dissection. Recognition of these variations is therefore important for reconstructive planning and identification of any risks with the procedure.

As mentioned previously, atypical anatomy or function of the thumb has a variety of downstream implications, ranging from clinical to daily functioning. As an integral component of hand biomechanics, patients with thumb hypoplasia or aplasia will likely require lifestyle and occupational modifications to perform everyday tasks or other additional activities effectively [22]. Understanding the different degrees of hypoplasia, the corresponding anatomical alterations, and potential additional clinical associations can help patients better understand their limitations and facilitate learning the adaptations best suited to them.

## Conclusions

Based on the findings presented in this case study, the authors conclude that this donor presentation is consistent with Grade V hypoplasia (aplasia), with unique variations in tendon insertions and compensation for neighboring anatomy. Due to a lack of evidence of surgical scarring in the area and abnormal forearm and hand musculature, the authors consider this to be of congenital origin, given the limited clinical history. This case study aims to contribute to the growing understanding of thumb hypoplasia and to provide deeper insight for individuals and clinicians experiencing similar situations, offering a greater understanding of the biomechanical, clinical, and lifestyle factors associated with it. Studying a case of thumb hypoplasia provides insight into the idea that gross, isolated malformations in patients can signal broader syndromic manifestations that may carry significant body-wide implications, including conditions such as VACTERL, Holt-Oram Syndrome, and thrombocytopenia-absent radius (TAR) syndrome. Recognizing the breadth of potential associations can help clinicians identify patients who may require additional workup to rule out potentially silent yet harmful additional impairments, like anemia, increased risk of malignancy, and bleeding risk.
